# CircGRB14 Inhibits Proliferation and Promotes Apoptosis of Granulosa Cells in Chicken Follicle Selection Through Sponging miR-12264-3p and miR-6660-3p

**DOI:** 10.3390/ijms26052214

**Published:** 2025-02-28

**Authors:** Huanqi Yang, Mengxiao Li, Beibei Zhang, Jinming Zhang, Yuxiang Shi, Tenghe Ma, Yanyan Sun

**Affiliations:** 1 College of Life Sciences and Food Engineering, Hebei University of Engineering, Handan 056038, China; yanghq947@163.com (H.Y.); li15133157636@163.com (M.L.); double0808@nwafu.edu.cn (B.Z.); 18034536145@163.com (J.Z.); hbyxshi@126.com (Y.S.); 2 State Key Laboratory of Animal Biotech Breeding, Institute of Animal Science, Chinese Academy of Agricultural Sciences, Beijing 100193, China

**Keywords:** chicken, circGRB14, miRNA, follicle selection, granulosa cell

## Abstract

The development and selection of ovarian follicles are essential for continuous egg production in chickens. Non-coding RNAs, particularly circular RNAs (circRNAs), play a critical regulatory role in follicle selection, a process heavily involving granulosa cells (GCs). In this study, we analyzed circRNA expression profiles in small yellow follicles (SYFs) and large yellow follicles (LYFs) of Taihang chickens using RNA sequencing. We identified 14,586 circRNAs, with 57 showing differential expression (DE-circRNAs) between SYFs and LYFs. Functional enrichment analysis revealed that these DE-circRNAs are involved in key biological processes, including signal transduction, cell membrane formation, and nuclear enzymatic regulation. We focused on circGRB14, a circRNA derived from the growth factor receptor-bound protein 14 (GRB14) gene, as a potential regulator of follicle selection. Using qPCR, CCK-8 proliferation assays, and Annexin V/PI apoptosis analysis, we demonstrated that circGRB14 inhibits GC proliferation and promotes apoptosis. In contrast, miR-12264-3p and miR-6660-3p, validated as direct targets of circGRB14 via Dual-Luciferase Reporter assays, exhibited opposing effects by promoting GC proliferation and inhibiting apoptosis. These findings highlight the circGRB14-miR-12264-3p/miR-6660-3p axis as a key regulatory mechanism in GC dynamics during follicle selection. This study provides novel insights into the functional interplay between circRNAs and miRNAs in avian follicle development, offering potential targets for improving egg production in poultry.

## 1. Introduction

Chickens are a significant economic resource in agriculture, particularly for egg production, which relies on the normal development and ovulation of follicles [1]. The performance of laying hens is intrinsically linked to the regular process of follicle selection, where one SYF (small yellow follicle) from the pool is chosen daily to mature into a LYF (large yellow follicle) [2,3]. This selection is a sophisticated and orchestrated biological event, modulated by various interacting pathways and cell types [4]. The ovarian follicle, the fundamental ovarian unit, comprises oocytes surrounded by GCs and theca cells (TCs) [5]. The GCs’ proliferation and differentiation are pivotal to follicle development, notably in the selection process [6]. Understanding the molecular underpinnings of follicle selection is essential for advancements in poultry genetics, breeding, and production.

Circular RNAs (circRNAs), once thought to be mere splicing artifacts, are now recognized as endogenous, circular molecules formed through back splicing [7,8,9]. They possess a covalently closed circular structure, lacking 5′ and 3′ ends, which makes them resistant to exonuclease degradation [10,11]. CircRNAs are categorized into exon circRNAs (ecircRNAs), intron circRNAs (ciRNAs), and exon–intron circRNAs (eiciRNAs) based on their origin [12]. MicroRNAs (miRNAs), approximately 22 nucleotides long, are non-coding small RNAs that are highly conserved across species and are key players in the post-transcriptional regulation of gene expression [13,14,15,16,17]. The advent of bioinformatics, molecular biology, and high-throughput sequencing has facilitated the identification of numerous circRNAs and miRNAs across various organisms [18,19,20].

Recent studies have revealed that circRNAs can act as miRNA sponges, implicating them in the regulation of numerous biological processes and cellular physiological changes [21,22]. For instance, circFGFR2 has been shown to promote the proliferation and differentiation of chicken skeletal muscle by sequestering miR-133a and miR-29b [23]. There is also growing evidence that circRNAs may play a crucial role in ovarian follicle development and selection. Abnormally expressed circRNAs have been suggested to contribute to porcine antral follicular atresia [24], and circSLC41A1 has been demonstrated to modulate the apoptosis of porcine follicular GCs through the competitive binding of miR-9820-5p [25]. A comparative study of circRNA expression profiles in GCs under red and white light conditions identified 2468 circRNAs, with 22 differentially expressed between the groups, including circRNA_0320 and circRNA_0185, which interacted with miR-143-3p to regulate the FSHR gene [26]. These findings highlight the potential roles of circRNAs in ovarian function. However, the specific role of circRNAs in follicle development in Taihang chickens, a Chinese domestic breed from Hebei Province, remains un-elucidated.

By RNA sequencing and bioinformatics analysis, we identified the differentially expressed circRNAs between SYFs and LYFs and constructed a circRNA–miRNA interaction network. circGRB14 is a circRNA derived from the growth factor receptor binding protein 14 (GRB14) gene, due to its potential regulatory role in GC dynamics. Moreover, circGRB14 was significantly different between large yellow follicles and small yellow follicles in the sequencing data, and we suspected that circGRB14 played an important role in follicle selection. Therefore, we focused on circGRB14. Our results showed that circGRB14 inhibited the proliferation of GCs and promoted their apoptosis, while its interacting miRNAs, miR-12264-3p and miR-6660-3p, counteracted these effects. These results provide new insights into the circRNA–miRNA regulatory axis in follicles.

## 2. Results

### 2.1. Overview of circRNAs

Six circDNA sequencing libraries were conducted from SYFs and LYFs of Taihang chickens, generating a total of 82.7 GB of 150 bp double-ended raw reads following ribosomal depletion and linear RNA digestion. Post-quality control, 550,889,180 clean reads were retained, with Q20 exceeding 95.05%, Q30 exceeding 88.13%, and GC content averaging around 48% (Table 1).

**Figure 1:**
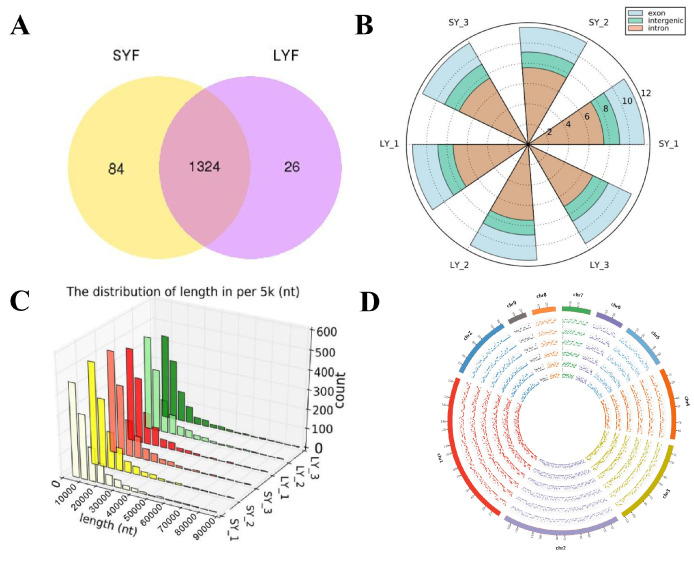
CircRNA expression profiles in chicken SYFs and LYFs. (**A**) Identification and differential expression analysis of circRNAs in SYFs and LYFs. The vertical bars represent the number of circRNAs identified in each group (SYFs, LYFs, or both). (**B**) Genomic origin distribution of the identified circRNAs. The vertical bars indicate the percentage of circRNAs derived from exonic, intronic, or exon–intron regions. (**C**) Length distribution of identified circRNAs. The vertical bars represent the frequency of circRNAs within specific length ranges (0–90,000 nucleotides). (**D**) Chromosome distribution of the identified circRNAs. The vertical bars show the percentage of circRNAs mapped to each chromosome in SYFs and LYFs.

A total of 1434 circRNAs were identified across the six follicle samples using two distinct methods, comprising 84 expressed in SYFs, 26 in LYFs, and 1324 in both (Figure 1A). The majority of these circRNAs were exonic, suggesting their origin from protein-coding exons (Figure 1B). The length of the identified circRNAs ranged from 0 to 10,000 nucleotides, as illustrated in Figure 1C.

The circRNAs in SYFs and LYFs were mapped to chromosomes 1 to 9 and Z of Taihang chickens, with a uniform distribution percentage across corresponding chromosomes. However, in LYFs, chromosome 1 showed the highest abundance of circRNAs, while chromosome 9 had the least (Figure 1D).

### 2.2. Expression Profiles of circRNAs During Follicular Selection

**Figure 2:**
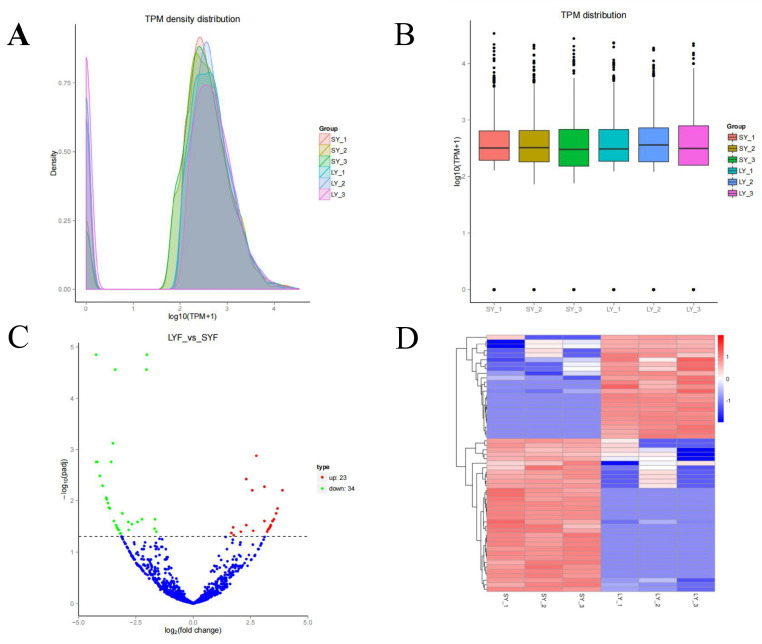
CircRNA expression dynamics during follicular selection. (**A**) Density distribution of circRNA TPM values across libraries. The vertical bars represent the density of circRNA expression levels (TPM values) in SYFs and LYFs. (**B**) Box plot representation of circRNA TPM values among the six libraries. The vertical bars (whiskers) indicate the range of TPM values, and the boxes represent the interquartile range (IQR). Groups compared: SYFs (small yellow follicles) and LYFs (large yellow follicles). (**C**) Volcano plot depicting differentially expressed circRNAs between SYFs and LYFs. The vertical axis represents the −log_10_ (adjusted *p*-value), and the horizontal axis represents the log_2_ (fold change). Red dots indicate significantly upregulated circRNAs, and blue dots indicate significantly downregulated circRNAs in LYFs compared to SYFs. (**D**) Hierarchical clustering of differentially expressed circRNAs in SYFs and LYFs. The vertical bars represent the expression levels (log_2_-transformed TPM values) of circRNAs, with red indicating high expression and blue indicating low expression. Groups compared: SYFs and LYFs.

CircRNA expression varies depending on tissue type and developmental stage [27], in this study, the TPM values of circRNAs showed two distinct peaks across all samples, indicating a consistent expression pattern during the chicken follicle selection stage (Figure 2A). These circRNAs were predominantly found within the TPM < 0.1 and TPM > 60. Variations in circRNA expression profiles were observed at different follicle developmental stages (Figure 2B).

### 2.3. Differentially Expressed circRNAs

To explore the role of circRNAs in follicular selection, we analyzed differential expression based on absolute fold changes ≥1.6 and adjusted *p* < 0.05. A total of 57 differentially expressed circRNAs were identified between SYFs and LYFs of Taihang chickens, with 23 upregulated and 34 downregulated in LYFs (Figure 2C). Hierarchical clustering revealed distinct expression patterns between SYF and LYF groups (Figure 2D).

### 2.4. Functional Annotation of DE-circRNAs

**Figure 3:**
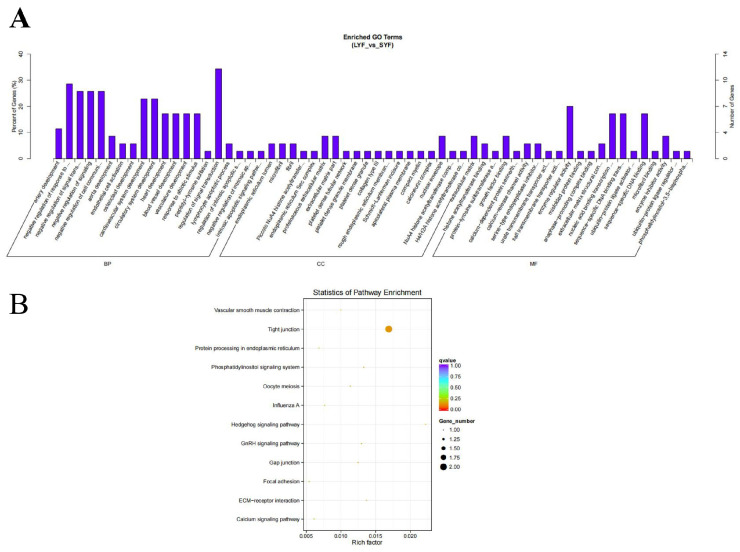
Functional enrichment analysis of differentially expressed circRNAs in chicken SYFs and LYFs. (**A**) GO enrichment histograms of circRNAs. The vertical bars represent the −log_10_ (*p*-value) of enriched GO terms related to signal transduction, nuclear membrane formation, and enzymatic regulation. (**B**) KEGG enrichment scatter plot of circRNAs. The vertical axis represents the −log_10_ (*p*-value), and the horizontal axis represents the enrichment factor. Groups compared: SYFs and LYFs.

Gene Ontology (GO) and Kyoto Encyclopedia of Genes and Genomes (KEGG) enrichment analyses were conducted on the host genes of differentially expressed circRNAs. The SYF and LYF groups were enriched in GO terms related to signal transduction, nuclear membrane formation, and enzymatic regulation (Figure 3A). Additionally, 12 KEGG pathways were significantly enriched in chicken SYFs and LYFs (Figure 3B).

### 2.5. CircGRB14 Identification and Expression in Chicken Follicles

**Figure 4:**
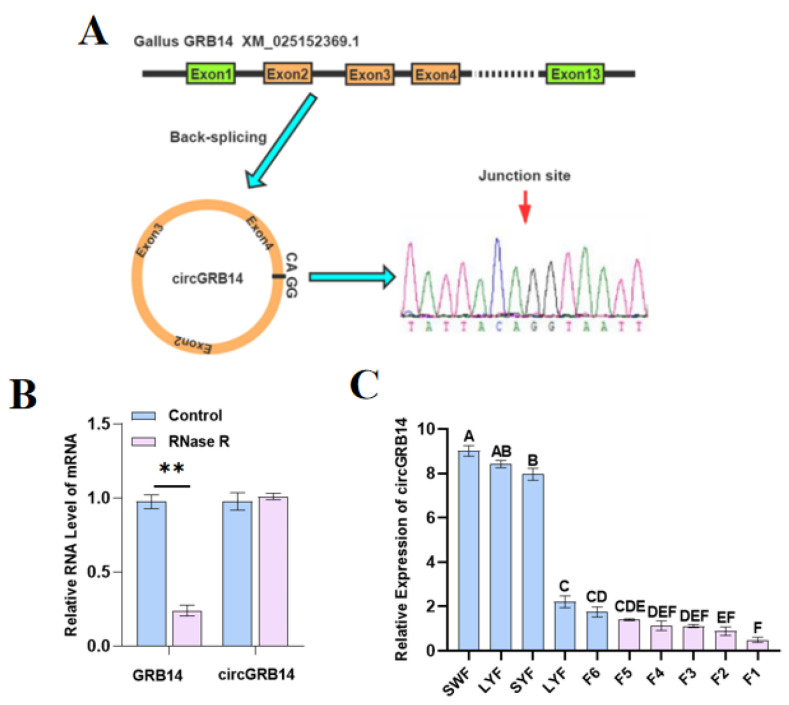
Characterization and expression profiling of chicken circGRB14. (**A**) Genomic localization of circGRB14 within the GRB14 gene and cyclization verification by PCR followed by Sanger sequencing. (**B**) Relative expression of circGRB14 and GRB14 in response to RNase R treatment versus NC. (**C**) Expression levels of circGRB14 across chicken follicles of varying diameters. Results were obtained from three biological replicates, and data are expressed as mean ± SEM. The different capital letters mean extremely significant difference (** *p* < 0.01).

CircGRB14 was found to be derived from exons 2 to 4 of the GRB14 gene (Figure 4A). Therefore, we named it circGRB14. Differential expression of the circGRB14 gene was observed through high-throughput sequencing, prompting an investigation into the role of circGRB14 in follicular development. RNase R treatment showed that circGRB14 was resistant to digestion, confirming its circular nature (Figure 4B). qRT-PCR revealed that circGRB14 expression decreased with increasing follicle diameter, particularly during the transition from SYFs to LYFs, with a significant difference (*p* < 0.01), suggesting a crucial role in follicle selection (Figure 4C).

### 2.6. CircGRB14 as an miRNA Sponge for miR-12264-3p and miR-6660-3p

**Figure 5:**
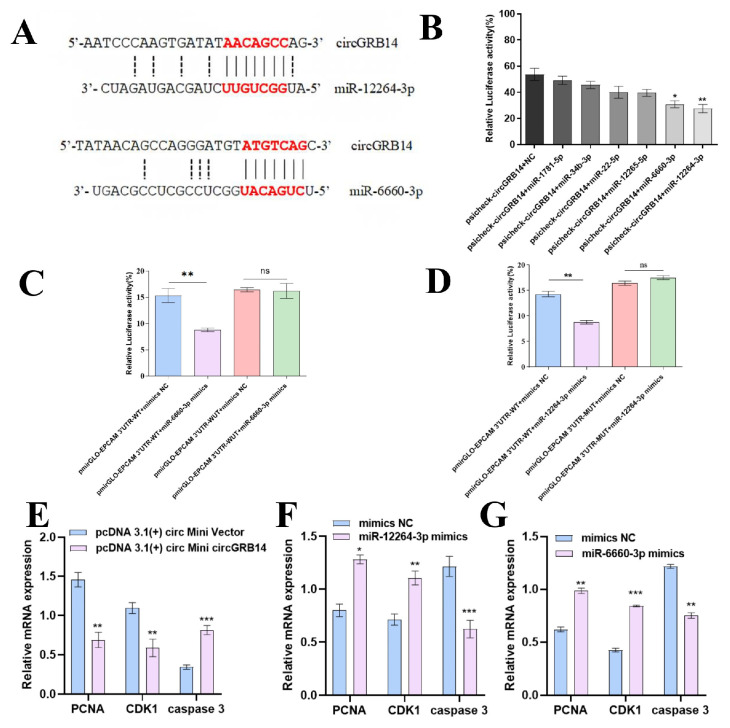
miRNA screening and validation of circular RNA targets using the Dual-Luciferase Reporter assay. (**A**) The interacting site of miR-12264-3p and the 6660-3p mion the sequence of circGRB14. (**B**) Dual-Luciferase Reporter assay of miR-12264-3, miR-12265-5p, miR-1781-5p, miR-22-5p, miR-34b-3p, miR-6660-3p, and circGRB14. (**C**) Dual-Luciferase Reporter assay of miR-12264-3 mimics or NC and circGRB14 wild-type or mutant plasmid. (**D**) Dual-Luciferase Reporter assay of miR-6660-3p mimics or NC and circGRB14 wild-type or mutant plasmid. (**E**–**G**) Relative mRNA levels of genes related to cell proliferation and apoptosis in chicken GCs after transfection with pcDNA 3.1(+) circ Mini circGRB14, miR-12264-3p mimics and miR-6660-3p mimics (* *p* < 0.05; ** *p* < 0.01; *** *p* < 0.001). ns—not significant.

We searched for the sequences of miR-12264-3p and miR-6660-3p in the miRBase sequence database and predicted their potential binding sites with circGRB14 (Figure 5A). A Dual-Luciferase Reporter assay confirmed that miR-12264-3p and miR-6660-3p, not miR-34b-3p, miR-12265-5p, miR-1781-5p, or miR-22-5p, reduced the fluorescence activity of the co-transfected circGRB14 Dual-Luciferase Reporter plasmid, indicating their regulatory role on circGRB14 (Figure 5B). Mutation of the target site in the plasmid abrogated the effect of these miRNAs, confirming the interaction of miR-12264-3p and miR-6660-3p with circGRB14 (Figure 5C,D).

### 2.7. CircGRB14′s Impact on GC Proliferation and Apoptosis

To assess the influence of circGRB14 on GC proliferation, we performed transfections with a circGRB14 overexpression vector into GCs. The Cell Counting Kit-8 (CCK-8) assay revealed no significant difference in absorbance at 24 and 48 h post-transfection. However, at 72 h, the overexpression of circGRB14 led to a significant reduction in GC proliferation compared to the NC (*p* < 0.01), suggesting that circGRB14 can inhibit cell proliferation.

**Figure 6:**
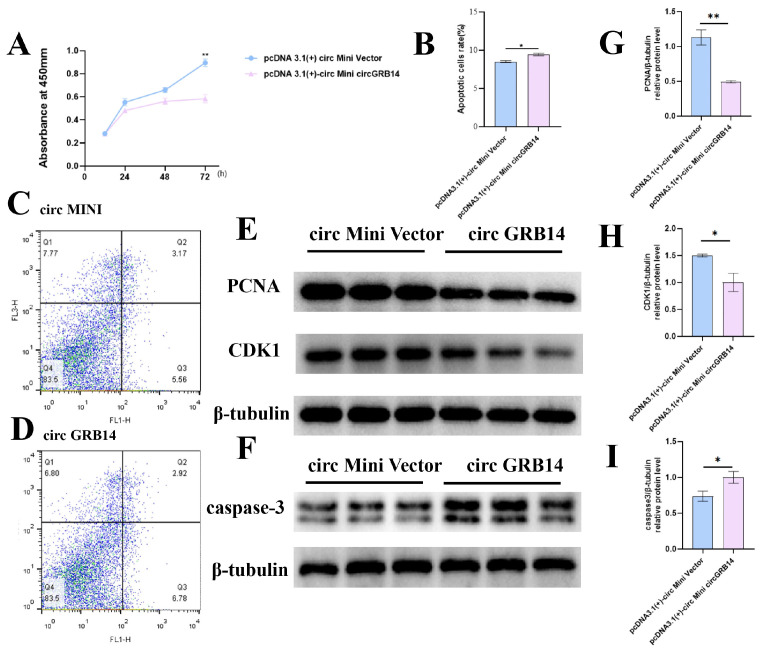
Impact of circGRB14 on chicken GC proliferation and apoptosis. (**A**) Influence of circGRB14 overexpression on GC proliferation, measured by absorbance at 24, 48, and 72 h post-transfection. (**B**–**D**) Effect of circGRB14 on GC apoptosis, with flow cytometry images and apoptosis rate analysis under transfection with pcDNA 3.1(+)-circ Mini circGRB14 or NC. (**E**,**F**) The levels of PCNA, CDK1, caspase-3, and β-tubulin proteins in GCs following transfection with pcDNA 3.1(+) circ Mini circGRB14. (**G**–**I**) PCNA, CDK1, and caspase-3 band intensities quantified by ImageJ (https://imagej.net/ij/notes.html accessed on 2 July 2022) and normalized against β-tubulin (* *p* < 0.05; ** *p* < 0.01).

To investigate the potential role of circGRB14 in the apoptosis of chicken GCs, we transfected cells with either pcDNA 3.1(+)-circ Mini circGRB14 or NC. Apoptosis was assessed 48 h post-transfection using Annexin V-FITC/PI staining followed by flow cytometry. The results revealed a significant increase in apoptotic cells in the circGRB14-transfected group compared to the control (*p* < 0.05), indicating that circGRB14 is implicated in the regulation of GC apoptosis. Western blot analysis also revealed that overexpression of circGRB14 suppressed the expression of PCNA and CDK1 proteins while promoting the expression of caspase-3 (Figure 6).

### 2.8. miR-12264-3p and miR-6660-3p’s Role in GC Proliferation and Apoptosis

**Figure 7:**
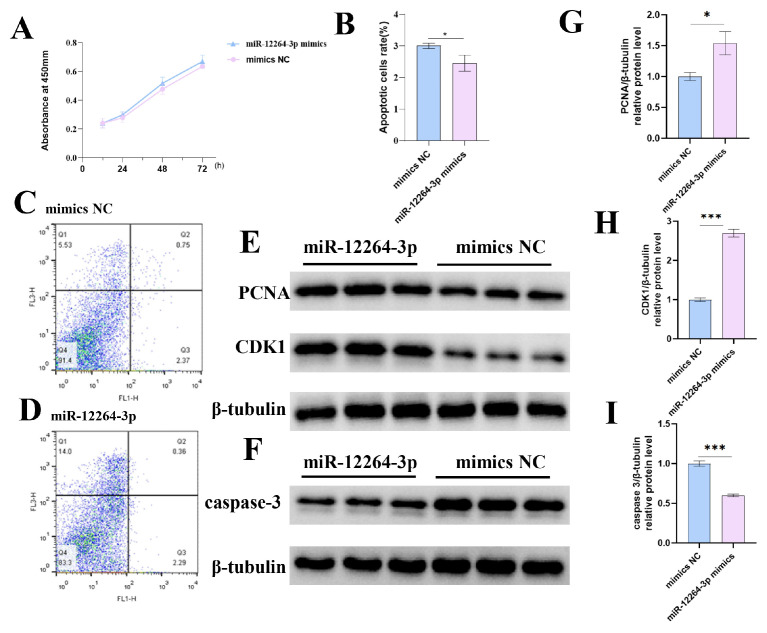
Impact of miR-12264-3p on chicken GC proliferation and apoptosis. (**A**) Influence of miR-12264-3p overexpression on GC proliferation, measured by absorbance at 24, 48, and 72 h post-transfection. (**B**–**D**) Effect of miR-12264-3p on GC apoptosis, with flow cytometry images and apoptosis rate analysis under transfection with miR-12264-3p mimics or NC. (**E**,**F**) The levels of PCNA, CDK1, caspase-3, and β-tubulin proteins in GCs following transfection with miR-12264-3p mimics. (**G**–**I**) PCNA, CDK1, and caspase-3 band intensities quantified by ImageJ (https://imagej.net/ij/notes.html accessed on 2 July 2022) and normalized against β-tubulin (* *p* < 0.05; *** *p* < 0.001).

**Figure 8:**
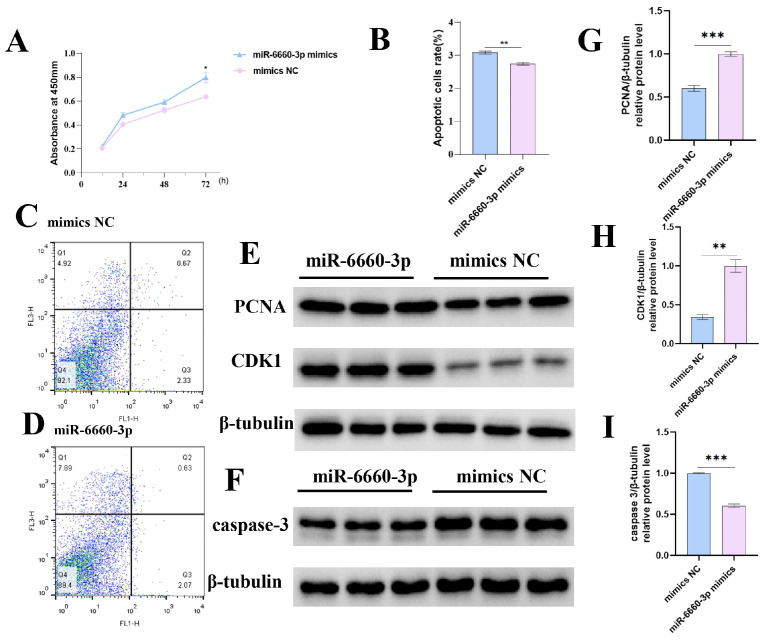
Impact of miR-6660-3p on chicken GC proliferation and apoptosis. (**A**) Influence of miR-6660-3p overexpression on GC proliferation, measured by absorbance at 24, 48, and 72 h post-transfection. (**B**–**D**) Effect of miR-6660-3p on GC apoptosis, with flow cytometry images and apoptosis rate analysis under transfection with miR-12264-3p mimics or NC. (**E**,**F**) The levels of PCNA, CDK1, caspase-3, and β-tubulin proteins in GCs following transfection with miR-6660-3p mimics. (**G**–**I**) PCNA, CDK1, and caspase-3 band intensities quantified by ImageJ (https://imagej.net/ij/notes.html accessed on 2 July 2022) and normalized against β-tubulin (* *p* < 0.05; ** *p* < 0.01; *** *p* < 0.001).

We also transfected GCs with mimics of miR-12264-3p, miR-6660-3p, and an NC to evaluate their effects on GC proliferation. The absorbance from the miR-12264-3p group increased over time, as shown in Figure 7A. While the absorbance in the miR-6660-3p group was higher at 24 and 48 h, it was not significantly different from that of the NC group; at 72 h, the miR-6660-3p group exhibited a significant increase in proliferation compared to the NC (*p* < 0.05) (Figure 7B). These flow cytometry results indicated that both miR-12264-3p and miR-6660-3p can inhibit apoptosis of GCs. The expression of the protein also corroborates this result (Figure 7 and Figure 8).

## 3. Discussion

The genetic mechanisms underlying poultry laying performance, particularly those regulating follicle selection, remain poorly understood. While environmental and nutritional factors are well documented, the role of non-coding RNAs, especially circRNAs, in follicle development has only recently gained attention [28,29,30,31]. This study aimed to address this gap by profiling circRNA expression in SYFs and LYFs of Taihang chickens. Using high-throughput sequencing, we identified 1434 circRNAs, with 57 showing differential expression during follicle selection. These findings align with previous studies suggesting that circRNAs are broadly involved in follicular development and cellular processes such as proliferation and differentiation [32,33]. However, our study is the first to systematically compare circRNA profiles before and after follicle selection in chickens, providing a foundation for understanding their regulatory roles.

One of the key findings of this study is the identification of circGRB14, a circRNA derived from the GRB14 gene, which is known to promote cell proliferation and differentiation through interactions with tyrosine kinase receptors [34,35,36]. Our results revealed that circGRB14 is highly expressed during follicle selection and negatively correlates with follicular diameter, suggesting a specific role in this process. This finding is consistent with Shen et al.’s prediction of dynamic, stage-specific circRNA expression in granulosa cells (GCs) [37]. Importantly, circGRB14’s function appears to be independent of its host gene, as circRNAs are known to exert regulatory effects distinct from their linear counterparts [38,39]. This highlights the potential of circGRB14 as a key regulator of follicle selection.

In the present study, we noted that circGRB14 expression was significantly lower in large yellow follicles than in small yellow follicles. Combined with its functional studies, circGRB14 may have a new mechanism and role in regulating follicle selection. Previous studies have shown that cell proliferation and apoptosis are regulated by cell cycle-related proteins (PCNA, CDK1 and CCND1) [40] and caspase family proteins [41]. The cyclin-dependent kinase (CDK) family is of crucial significance in governing cell cycle transitions. These kinases are activated by diverse cyclins at precise temporal points, thereby propelling the progression of the cell cycle. CDKs are indispensable for cell proliferation across nearly all animal species [42]. In this study, we selected two cell proliferation genes, including PCNA and CDK1, and a cell proliferation-related gene, caspase-3. Overexpression of circGRB14 reduced the expression levels of cell proliferation-related genes (PCNA and CDK1), which was consistent with the results obtained by CCK-8. The mRNA and protein expression levels of pro-apoptotic gene caspase-3 were increased, and the number of apoptotic cells detected by flow cytometry was increased.

A critical consideration in this study is the evidence supporting the hypothesis that circGRB14 functions as an miRNA sponge. To elucidate the regulatory mechanisms of circGRB14, we identified and validated two targeting miRNAs, miR-12264-3p and miR-6660-3p, using Dual-Luciferase Reporter assays. Functional experiments demonstrated that circGRB14 inhibits GC proliferation and promotes apoptosis, while miR-12264-3p and miR-6660-3p exhibit opposing effects. These results are supported by studies on other circRNAs, such as circ_PLXNA1 and circZNF609, which also regulate cell differentiation and proliferation [43,44]. Our findings suggest that the circGRB14-miR-12264-3p/miR-6660-3p axis plays a critical role in modulating GC dynamics during follicle selection, providing new insights into the molecular regulation of ovarian function.

While bioinformatics predictions (e.g., TargetScan and miRanda analysis) and the observed counterregulatory effects of circGRB14 overexpression on granulosa cell proliferation and cytoplasmic/mitochondrial apoptosis relative to miR-12264-3p/miR-6660-3p inhibition (as evidenced by Caspase-3 activation) are consistent with a sponge adsorption mechanism, we acknowledge that direct experimental validation of miRNA–circRNA interactions is required to establish causality. This will be done in further studies. Our current findings are consistent with methodological advances in basic circRNA research, such as Memczak et al. [45], who initially proposed CDR1as as an miR-7 sponge through bioinformatics and functional antagonism, and subsequently performed RNA pulldown assays and Ago2 immunoprecipitation. The correlation patterns reported here provide a solid foundation for future studies to mechanistically analyze the circrNA-mediated sponge effect.

In conclusion, this study provides the first comprehensive analysis of circRNA expression during follicle selection in Taihang chickens, identifying circGRB14 as a key regulator of GC proliferation and apoptosis. The circGRB14-miR-12264-3p/miR-6660-3p axis represents a novel regulatory mechanism in follicle development, offering new avenues for improving poultry reproduction. These findings not only enhance our understanding of circRNA biology but also lay the groundwork for practical applications in animal production and reproductive medicine.

## 4. Materials and Methods

### 4.1. Ethics Statement

All procedures conducted within this study were reviewed and approved by the Animal Welfare Department of the Institute of Animal Science at Hebei University of Engineering (Approval No. BER-YXY-2024050). Efforts were made to quantify and minimize animal distress during all experimental procedures.

### 4.2. Chicken Follicle Harvesting

Taihang chickens were sourced from the conservation base of Hebei Tiankai Poultry Industry Technology Co., Ltd. (Handan, China) The birds were housed individually and maintained on a uniform diet and environmental conditions until they reached 43 weeks of age, at which point they were humanely euthanized. The ovaries weare subsequently extracted and washed with PBS (Invitrogen, Carlsbad, CA, USA). The SYFs and LYFs were collected for circRNA sequencing. The SYFs were specifically utilized for GC isolation. A total of 18 chickens were used, with 6 chickens per group, for SYF and LYF collection.

### 4.3. CircRNA Sequencing and Identification

Total RNA from SYF and LYF samples of three individuals was isolated using TRIzol reagent (Invitrogen, Carlsbad, CA, USA). RNA quality and concentration were assessed through gel electrophoresis, spectrophotometry (NanoDrop 2000, Thermo Fisher Scientific, Waltham, MA, USA), and fluorometry (Qubit 3.0, Thermo Fisher Scientific, Waltham, MA, USA). For circRNA library construction, ribosomal RNA was depleted using the Epicentre Ribo-zero™ Kit (Epicentre, Madison, WI, USA), followed by ethanol precipitation to purify rRNA-free residue. Linear RNA was digested with RNase R (Epicentre, Madison, WI, USA), and sequencing libraries were constructed using the NEBNext^®^ UltraTM Directional RNA Library Prep Kit (NEB, Ipswich, MA, USA). PCR products were purified using the Vazyme FastPure Gel DNA Extraction Mini Kit (Vazyme, Nanjing, China), and the quality of the library was assessed using the Agilent Bioanalyzer 2100 system (Agilent Technologies, Santa Clara, CA, USA). Sequencing was performed on an Illumina Hiseq 4000 platform (Illumina, San Diego, CA, USA), yielding 150 bp paired-end reads. Quality control was ensured by calculating Q20, Q30, and GC content. Reads were aligned to the reference genome using bowtie2 (v2.0.6, http://bowtie-bio.sourceforge.net/bowtie2/index.shtml, accessed on 2 July 2022) [46], and circRNAs were identified with Find_CIRC (v1.1) [45] and CIRI2 (v1.2) [47]. Circos (v0.62-1) mapping was utilized to examine read distribution across chicken chromosomes [48].

### 4.4. Identification of Differentially Expressed circRNAs

CircRNA expression levels were quantified and normalized by Transcripts Per Million (TPM) [49]. DESeq2 (v1.10.1) [50] was applied for differential analysis, identifying circRNAs with absolute fold changes ≥1.6 and adjusted *p* < 0.05. Each group consisted of three biological replicates, and the analysis was repeated twice to ensure reproducibility.

### 4.5. GO and KEGG Enrichment Analysis

GO enrichment analysis for host genes of differentially expressed circRNAs was conducted using the GOseq R program (v2.12) with gene length bias correction. GO terms with adjusted *p* < 0.05 were considered significantly enriched. KEGG pathway enrichment for differentially expressed genes or circRNA host genes was assessed using KOBAS software (v2.0) [51]. Adjusted *p* < 0.05 indicated significant enrichment. The analysis was performed on three biological replicates per group.

### 4.6. CircGRB14 Validation and qRT-PCR

CircGRB14, derived from exons 2, 3, and 4 of the GRB14 gene, was selected for further study. Total RNA from ovaries and follicles of varying grades was isolated for cyclization identification of circGRB14. cDNA was synthesized using the HiScript III 1st Strand cDNA Synthesis Kit (+gDNA wiper) (Vazyme, Nanjing, China). PCR-circGRB14 primers were designed to amplify the circRNA splicing site [52], and PCR products were confirmed by agarose gel electrophoresis and sequenced (Table 2). For qRT-PCR, SYF total RNA was digested with RNase R, and qRT-PCR was conducted to evaluate circGRB14 and GRB14 expression variations. The 18s rRNA and GAPDH genes served as internal controls. ChamQTM Universal SYBR^®^ qPCR Master mix (Vazyme, Nanjing, China) was used for qPCR, and melting curves were analyzed to confirm primer specificity. The 2^−ΔΔCT^ method was employed to calculate the relative expression [53]. Each experiment was performed in triplicate.

### 4.7. Plasmid Construction and Dual-Luciferase Reporter Assay

Full-length circGRB14 was synthesized and cloned into the psicheck2 vector (Promega, Madison, WI, USA) to create a Dual-Luciferase plasmid. The seed region of miRNA (usually nucleotides 2–7 or 2–8) was utilized to match with the circRNA sequence.

The complementary pairing of the seed region is key to miRNA binding to its target. The binding miRNAs of circGRB14 were selected by searching the matched seed sequence. MiR-12264-3p, miR-12265-5p, miR-1781-5p, miR-22-5p, miR-34b-3p, and miR-6660-3p were predicted to target circGRB14. Mutations were introduced into the target regions of miR-12264-3p and miR-6660-3p within circGRB14 to construct mutant plasmids. Transfection of 293T cells was performed in 96-well plates using GenXPIII Transfection Reagent (Probe Gene, Xuzhou, China). Dual-Luciferase activity was measured after 48 h using the Dual-Luciferase^®^ Reporter Assay System (Promega, Madison, WI, USA). Each experiment was performed in triplicate, with positive controls (plasmid without miRNA) and negative controls (plasmid with scrambled miRNA).

### 4.8. GC Isolation and Culture

GCs were isolated from SYFs using a method adapted from Gilbert et al. [54]. Cell viability and density were assessed using Trypan blue staining (Sigma-Aldrich, St. Louis, MO, USA), and cells were cultured in M199 media (Gibco, Grand Island, NY, USA) with 10% fetal calf serum (Gibco, Grand Island, NY, USA) at 37 °C in a CO_2_ incubator (Thermo Fisher Scientific, Waltham, MA, USA). Each experiment was performed with three biological replicates.

### 4.9. Cell Transfection

CircGRB14 was cloned into the pcDNA 3.1(+)-circ Mini Vector (Invitrogen, Carlsbad, CA, USA) with NheI\ApaI enzyme sites. Transfection of GCs was carried out using Lipofectamine 3000 (Invitrogen, Carlsbad, CA, USA) with pcDNA 3.1(+)-circ Mini Vector, pcDNA 3.1(+)-circ Mini circGRB14, miRNA mimics, and mimics NC. Each transfection experiment was performed in triplicate.

### 4.10. Cell Proliferation Assay

The Cell Counting Kit-8 (CCK-8) (Dojindo, Kumamoto, Japan) was used to assess GC proliferation. Chicken GCs were plated in 96-well plates, and CCK-8 reagent was added post-transfection at 24, 48, and 72 h. Absorbance at 450 nm was measured using a Multiskan SkyHigh microplate spectrophotometer (Thermo Fisher Scientific, Waltham, MA, USA). Each experiment was performed in triplicate, with positive controls (cells without transfection) and negative controls (cells transfected with empty vector).

### 4.11. Cell Apoptosis Assay

Apoptosis was measured by flow cytometry with the Annexin V-FITC/PI Apoptosis Detection Kit (Vazyme, Nanjing, China). Cells were processed and stained according to the manufacturer’s instructions, and data were analyzed using FlowJo_V10 software (BD Biosciences, Bedford, MA, USA). Each experiment was performed in triplicate, with positive controls (cells treated with apoptosis inducer) and negative controls (untreated cells).

### 4.12. Western Blot

Isolated primary chicken granulosa cells were transfected 48 h after transfection, and then lysed in buffer (Beyotime, Shanghai, China) supplemented with protease inhibitors (Roche Diagnostics, Basel, Switzerland). For immunoblot analysis, protein samples (30 μg) were mixed with loading buffer (50 mM Tris HCl [pH 6.8], 2% SDS, 100 mM DTT, 10% glycerol, and 0.1% bromphenol blue), boiled, applied to 10% SDS-polyacrylamide gels, and subjected to electrophoresis. Immunoblot analysis was performed as previously described, using primary Abs against CDK2 (1:1000; 19532-1-AP, Proteintech, Wuhan, China), PCNA (1:1000; 19532-1-AP, Proteintech, Wuhan, China), caspase-3 (1:1000; 25128-1-AP, Proteintech, Wuhan, China), and β-tubulin (1:2000; 10094-1-AP, Proteintech, Wuhan, China). After washing, membranes were incubated with corresponding secondary antibodies (1:2000; SA00001-2, Proteintech, Wuhan, China), and bands were visualized by immunofluorescence using an enhanced chemiluminescence detection system (Thermo Fisher Scientific, Waltham, MA, USA). Densitometric analysis was as described previously; the intensity of the protein band was divided by that of the β-tubulin, and this ratio was then expressed relative to that of the untreated control, which was set at 100%. In all instances, density values of bands were corrected by subtraction of the background values. Each Western blot experiment was performed in triplicate, and the figures represent all obtained results.

### 4.13. Statistical Analyses

Data are presented as mean ± SEM. Statistical significance was determined at *p* < 0.05 using SPSS 20.0 Software (IBM, Armonk, NY, USA), and graphs were generated with GraphPad Prism (v8.0.2) (GraphPad Software, San Diego, CA, USA). Each experiment was performed with at least three biological replicates.

## 5. Conclusions

**Figure 9:**
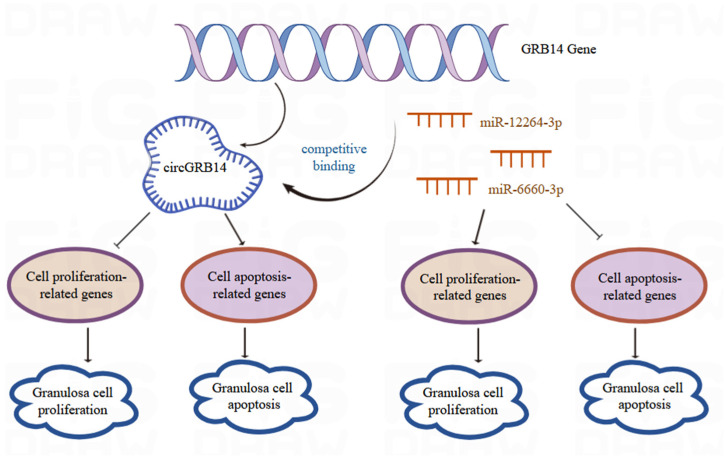
Mechanism diagram of CircGRB14 inhibiting proliferation and promoting apoptosis of chicken follicle granulosa cells by sponging miR-12264-3p and miR-6660-3p.

Our study represents a pioneering effort in characterizing the dynamic expression patterns of circRNAs in Taihang chickens in relation to follicular selection. We confirmed the presence of the circGRB14 and its differential expression across various follicle grades. Notably, forced expression of circGRB14 resulted in suppressed cell proliferation and enhanced apoptosis, whereas the targeted miRNAs miR-12264-3p and miR-6660-3p stimulated proliferation and mitigated apoptosis (Figure 9). The findings of this study lay a foundational theoretical framework for understanding the intricate biological roles of circRNAs in avian follicular development.

## Figures and Tables

**Table 1:** Sequencing results and quality evaluation in six libraries.

Sample	raw_reads	clean_reads	clean_bases	error_rate (%)	Q20 (%)	Q30 (%)	GC_content (%)
SYF_1	96589128	81847658	12.28G	0.02	96.86	92.22	50.21
SYF_2	103783026	98453096	14.77G	0.02	96.35	91.01	48.52
SYF_3	95081398	90236062	13.54G	0.02	96.41	91.12	49.65
LYF_1	95699154	89900182	13.49G	0.02	95.35	89.12	48.18
LYF_2	99396460	93902260	14.09G	0.02	96.12	90.62	46.96
LYF_3	103617614	96549922	14.48G	0.03	95.05	88.13	47.23

**Table 2:** Primers for circRNA identification and qRT-PCR.

Primer	Sequence	Annealing Temp (°C)	Product Size (bp)
PCR-circGRB14	F: 5′-AACTGTAAAACACTCTGGGA-3′ R: 5′-CTAAGCCTGTATGAGTAAGT-3′	60	210
circGRB14	F: 5′-CAAACTGGGGGATGGAAGAG-3′ R(RT): 5′-CAGTTGCATTGGTCTCACTTGAA-3′	59	132
*GRB14*	F: 5′-GGAGGAACCAGAGACCTGAAACAAC-3′ R: 5′-GCAGCCACTTCTACCTTGATACGG-3′	60	141
18s rRNA	F: 5′-TAGTTGGTGGAGCGATTTGTCT-3′ R: 5′-CGGACATCTAAGGGCATCACA-3′	60	169
*GAPDH*	F: 5′-CTGTGCCCATCTATGAAGGCTA-3′ R: 5′-ATTTCTCTCTCGGCT-GTGGTG-3′	60	139

## Data Availability

The original contributions presented in this study are included in the article. Further inquiries can be directed to the corresponding authors.
